# The duodenal microbiota is compartmentalized and clinically stable yet rapidly responsive to nutrient exposure

**DOI:** 10.1080/19490976.2026.2657053

**Published:** 2026-04-18

**Authors:** Jacqueline Wyss, Stefan Baehler, Jasmin Ferracini, Dino Kroell, Anne Marie Rossier, Niklas Krupka, Benjamin Misselwitz, Bahtiyar Yilmaz, Reiner Wiest

**Affiliations:** a Department of Visceral Surgery and Medicine, Bern University Hospital, University of Bern, Bern, Switzerland; b Maurice Müller Laboratories, Department for Biomedical Research, University of Bern, Bern, Switzerland; c Graduate School for Health Sciences, University of Bern, Bern, Switzerland

**Keywords:** Small intestine, microbiota, duodenum, SIBO, 16S rRNA gene sequencing, intraluminal fat challenge

## Abstract

The duodenum is one of the most nutrient-exposed and immunologically active regions of the human gastrointestinal tract, yet its microbial ecology and short-term responses to dietary stimuli remain poorly defined. Most studies rely on fasting luminal aspirates, which fail to capture mucosa-associated communities and miss rapid ecological shifts during nutrient exposure, limiting insight into the spatial organization and dynamic behavior of the upper small intestinal microbiota (SIM). To address these limitations, we performed a compartment-resolved analysis of the duodenal microbiota in 94 individuals, including healthy controls, patients with obesity before and after bariatric surgery, individuals with IBS, and subjects with other SIBO-associated risk states. Paired luminal aspirates and mucosal biopsies were obtained during upper endoscopy; bacterial load was quantified by culture, and the community structure was assessed using 16S rRNA gene sequencing and PICRUSt-based pathway inference. In addition, healthy volunteers underwent a controlled intraluminal fat challenge with dense serial duodenal sampling over 180 min to resolve short-term nutrient-driven dynamics. Across all participants, the anatomical niche emerged as the dominant ecological determinant. Mucosal communities displayed higher species richness, broader phylogenetic representation, and distinct beta-diversity signatures compared with luminal aspirates, which were narrowly dominated by *Streptococcaceae*. Under fasting conditions, SIM remained remarkably stable across obesity, IBS, and culture-defined SIBO, with only minor taxonomic differences in SIBO-positive individuals. In contrast, acute nutrient exposure triggered rapid microbial blooming, increased culture positivity, and a transient rise in species richness within 30–60 min, revealing a highly responsive ecosystem not captured by fasting samples. Together, these findings show that the defining feature of the human duodenal microbiota is not disease-associated dysbiosis under fasting conditions, but rather a conserved spatial architecture coupled with rapid, nutrient-driven ecological plasticity, highlighting the dynamic and compartmentalized nature of the upper small intestinal microbiome.

## Introduction

Despite being the site where digestion begins and host-microbe interactions are most immediate, the upper small intestine remains one of the least characterized microbial ecosystems in the human gut. It is shaped by steep physicochemical gradients driven by rapid nutrient flux, fluctuating oxygen tension near the mucosa, and constant exposure to host-secreted digestive and immunological factors.^1-3^ These conditions differ fundamentally from the more stable, fermentation-dominated colonic niche.^4^ However, most microbiome research continues to rely on stool, capturing only distal communities and providing limited insight into the proximal gut.^5-7^ As a result, fundamental questions about the organization, stability, and functional behavior of the duodenal microbiota remain unresolved.

Progress has been largely constrained by sampling limitations. Because the duodenum is accessible only by endoscopy, most human studies rely on fasting luminal aspirates.^3^^,^^5^ While aspirates provide a useful snapshot of luminal contents, they fail to capture mucosa-associated communities residing within structured microenvironments shaped by oxygen diffusion, nutrient exchange, epithelial metabolites, and antimicrobial peptides.^1^^,^^8-10^ The mucosal surface sustains a host-regulated niche favoring adhesive, mucin-degrading, and microaerophilic taxa, whereas the lumen is characterized by rapid flow, intermittent substrate pulses, and residual gastric acidity, conditions that select for fast-growing aerotolerant organisms such as *Streptococcaceae.*^3^^,^^7^^,^^11^^,^^12^ Only a limited number of studies have performed truly paired aspirate-biopsy sampling from the same duodenal site,^7^^,^^13^^,^^14^ leaving the extent and clinical consistency of compartmentalization incompletely defined.

Small intestinal bacterial overgrowth (SIBO) has long served as the dominant clinical framework for interpreting abnormalities in the proximal small intestine.^15^^,^^16^ Traditionally defined by elevated bacterial counts in fasting duodenal aspirates, SIBO is frequently suspected in patients with motility disturbances, postprandial symptoms, or unexplained upper gastrointestinal discomfort.^17^^,^^18^ Obesity and type 2 diabetes, for instance, are commonly associated with delayed transit and increased rates of culture-defined SIBO,^19-22^ and many individuals with IBS are evaluated for overgrowth due to overlapping symptom profiles.^23-26^ However, these associations largely derive from fasting aspirates cultures and may reflect clinical practice patterns rather than ecological restructuring. Culture thresholds established decades ago are inconsistently applied, do not reflect mucosal communities, and fail to capture rapid, substrate-driven microbial fluctuations.^27^ Sequencing-based studies have revealed discrepancies between culture positivity and community composition,^28-30^ underscoring the limited ecological resolution of current diagnostic constructs.

Recent work has advanced the field by systematically mapping the small-intestinal microbiota using endoscopic sampling, demonstrating that luminal communities differ markedly from stool and vary along small-bowel segments,^5^ while also highlighting the technical challenges inherent to low-biomass aspirates.^3^ In parallel, disease-focused studies such as those in celiac disease have shown that the sampling location within the duodenum can strongly influence the observed microbial composition and predicted function, emphasizing the need for anatomical standardization and careful interpretation of biogeographic signals.^31^ Together, these studies establish that both the sampling depth and anatomical site are critical determinants of small-intestinal microbiome profiles. However, most available datasets either focus on disease-specific cohorts or segmental variation and do not systematically integrate paired mucosal and luminal sampling with controlled physiological perturbation within the same study design. However, two central questions remain unresolved: first, whether disease labels and culture-defined SIBO reflect major ecological reorganization once mucosal and luminal niches are examined in parallel; and second, whether fasting sampling accurately represents the functional dynamics of a system defined by rapid nutrient exposure.

The physiological dimension is particularly underexplored. Nearly all human small-intestinal microbiome studies are performed in the fasted state,^14^ despite the fact that the duodenum is characterized by rapid, repeated substrate influx. Nutrient arrival alters oxygen gradients,^10^ reshapes substrate availability,^32-34^ and modulates epithelial signaling pathways,^35^ generating microenvironmental shifts that may reorganize microbial communities within minutes. While rapid diet-induced changes are well documented in the fecal microbiota and model systems, high-resolution temporal data from the human upper small intestine remain scarce. Consequently, fasting aspirates may systematically underestimate transient but physiologically relevant microbial states, including nutrient-driven blooms that influence postprandial symptoms or upper-gut dysbiosis.

We hypothesized that the anatomical niche and physiological state constitute primary organizing axes of duodenal microbial ecology, potentially outweighing disease labels under fasting conditions while revealing rapid ecological plasticity during nutrient exposure. To test this hypothesis, we performed compartment-resolved profiling of the duodenal microbiota across clinically relevant phenotypes using paired luminal aspirates and mucosal biopsies collected from a standardized site in the second portion of the duodenum during upper endoscopy. The bacterial load was quantified by culture, and the microbial diversity and community structure were characterized using 16S rRNA gene sequencing. In a separate healthy volunteer cohort, we integrated a controlled intraduodenal fat challenge with dense serial sampling over 3 h to capture short-term postprandial dynamics.

This combined design enables simultaneous evaluation of spatial architecture (mucosa versus lumen), clinical context (obesity, IBS, bariatric surgery, and SIBO-associated risk states), and acute physiological perturbation. By integrating compartmental resolution with controlled nutrient exposure, this study provides an ecological framework for interpreting culture-defined SIBO and symptom burdens beyond the constraints of fasting aspirates, and reveals a duodenal ecosystem characterized by conserved compartmentalization and rapid, reversible nutrient-responsive plasticity.

## Methods

### Study cohorts and ethical approval

**Figure 1.**
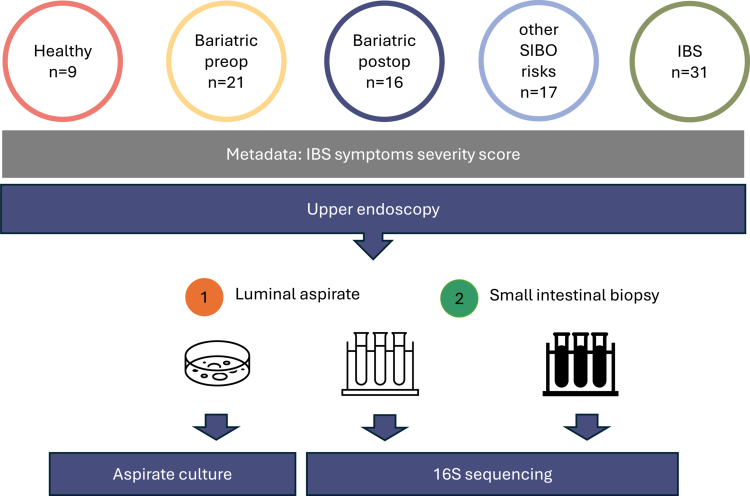
**Study design and sampling workflow.** The flowchart illustrates a study design and sampling workflow. The study included five participant groups: healthy controls (n = 9), bariatric preoperative individuals (n = 21), bariatric postoperative individuals (n = 16), individuals with other SIBO-associated risk factors (n = 17), and patients with IBS (n = 31). IBS symptom severity scores were recorded as part of the clinical metadata. Following upper endoscopy, paired luminal aspirates and small intestinal biopsies were collected. Aspirates were subjected to both culture-based analysis and 16S rRNA gene sequencing, whereas biopsy samples were analyzed by 16S rRNA gene sequencing. This design enabled the integrated profiling of upper small-intestinal microbial communities across luminal and mucosal niches.

Subjects aged 18–85 y undergoing esophagogastroduodenoscopy (EGD) for standard-of-care evaluation were prospectively recruited at the Department of Visceral Surgery and Medicine, Bauchzentrum, Inselspital, Bern, Switzerland. Additionally, healthy volunteers for the nutrient-challenge experiment were recruited. Figure 1 and Supplementary Figure S1 provide an overview of the study design, cohort structure, and sampling workflow. Ethical approval was granted by the Cantonal Ethics Committee of Bern (KEK 2020-02116, KEK 2017-00923, KEK 2016-00571), and all participants provided written informed consent.

Demographic variables (age, sex, BMI), medication profiles, dietary factors, and SIBO-associated risk factors (e.g., PPI use, gastric surgery) were collected using standardized questionnaires prior to the SIBO assessment. The data were anonymized and entered into the Bern REDCap database hosted by the Clinical Trial Unit.

#### Healthy controls

The healthy control group consisted of non-obese adults (BMI  <25 kg/m²) undergoing EGD for indications unrelated to gastrointestinal disease. Individuals were free of conditions known to affect the intestinal microbiota, were medication-free, and reported no smoking, alcohol, or substance use. The exclusion criteria included age  <18 y, pregnancy or breastfeeding, recent antibiotic exposure (<4 weeks), anticoagulation or bleeding disorders, contraindications to endoscopy, major systemic illness, and inability to provide informed consent.

#### IBS and SIBO-associated risk cohorts

Patients presenting with IBS or symptoms suggestive of small intestinal dysbiosis were eligible for inclusion. This cohort comprised individuals with a clinical diagnosis of IBS (with or without a positive breath test), patients exhibiting symptoms or comorbidities commonly associated with SIBO, including scleroderma, diabetic neuropathy, amyloidosis, fibromyalgia, or altered intestinal anatomy such as diverticula, and individuals with a positive lactulose breath test, defined as a rise of ≥20 ppm hydrogen or ≥8 ppm methane within 60 min. Additional eligible participants included those taking medications that affect gastrointestinal motility or gastric acid secretion, as well as symptomatic patients reporting bloating, flatulence, or diarrhea without an alternative diagnosis. IBS symptom severity was quantified using the validated IBS Severity Scoring System (IBS-SSS), which ranges from 0 to 500, with scores  >300 indicating severe disease.

#### Bariatric cohorts

Two bariatric subgroups were investigated. The pre-operative cohort consisted of obese adults (BMI ≥35 kg/m²) scheduled for bariatric surgery who underwent routine EGD within two months prior to intervention. The post-operative cohort comprised individuals who had undergone Roux-en-Y gastric bypass or sleeve gastrectomy and subsequently developed symptoms suggestive of SIBO, including bloating, abdominal distension, discomfort or pain, diarrhea or steatorrhea, or inadequate weight loss. Post-operative patients with culture-confirmed SIBO received a standardized 10-d regimen of selective intestinal decontamination with gentamicin/polymyxin administered four times daily. A follow-up EGD was performed within two weeks after the completion of antibiotic therapy.

#### Endoscopic sampling approaches for duodenal aspirates and mucosal biopsies

EGDs were performed under conscious sedation using propofol (1%). A pediatric colonoscope (PCF-190/180) or gastroscope (GIF-H190/180) was advanced to the third portion of the duodenum (D3) under direct visualization with minimal CO₂ insufflation. All luminal aspirates and mucosal biopsies were systematically obtained from this standardized D3 location to minimize segmental variation along the duodenum. Luminal aspirates were collected via a sterile catheter (Gastro-Bacterio-Kath, Vygon) using a 20 ml syringe (minimum volume 0.5 ml) and immediately split for culture and sequencing. Mucosal biopsies were collected using sterile forceps and transferred into pre-weighed sterile tubes.

#### Microbiological culture

Aspirates were serially diluted (1:10–1:10^4^) in balanced salt solution and plated on Wilkins-Chalgren agar containing 5% sheep blood (anaerobic) and LB agar (aerobic). The plates were incubated at 37 °C for 48–72 h, with extended incubation for negative cultures. The colony counts were expressed as CFU/ml. SIBO was defined as ≥10³ CFU/ml.

#### DNA extraction

DNA was extracted from luminal aspirates and mucosal biopsies using optimized protocols tailored to each sample type. Stool and luminal content were processed with the QIAamp DNA Stool Kit (Qiagen), whereas biopsies were extracted using the AllPrep DNA/RNA Mini Kit (Qiagen).

For luminal content and stool, approximately 100 mg of material was aliquoted into 2 ml microfuge tubes and stored at −20 °C until extraction. The samples were homogenized in 500 µL of Buffer ASL using a Retsch TissueLyser at 30  Hz for 3 min, followed by two additional lysis steps at 95 °C. To enhance the disruption of Gram-positive bacteria, the samples were incubated with 200 µL lysozyme-containing Gram-positive lysis buffer (20 mg/ml lysozyme, 20 mM Tris-HCl pH 8.0, 2 mM EDTA, and 1.2% Triton X-100). After the addition of 500 µL of Buffer ASL, the remainder of the manufacturer’s protocol was followed. DNA was eluted in 30 µL of RNase-free water and stored at −20 °C.

Biopsy specimens were collected into RNAlater (Sigma-Aldrich) and stored at −80 °C prior to extraction. Each sample was homogenized in 600  µL of Buffer RLT Plus supplemented with *β*-mercaptoethanol and a metal bead using the Retsch TissueLyser (30  Hz, 3 min). The lysates were centrifuged for 3 min at maximum speed, transferred to AllPrep DNA spin columns, washed with AW1 and AW2 buffers, and eluted in 20–30 µL of EB buffer. DNA quantity and purity were assessed via NanoDrop (Thermo Scientific).

## 16S rRNA gene amplification and sequencing

Bacterial community profiling targeted the V5‒V6 hypervariable regions of the 16S rRNA gene.^36^ PCR amplification was performed using KAPA HiFi HotStart ReadyMix (Roche) with 200‒1000 ng of template DNA and barcoded, bacteria-specific primers: forward: 5′-CCATCTCATCCCTGCGTGTCTCCGACTCAGC-barcode-ATTAGATACCCYGGTAGTCC-3′ and reverse: 5′-CCTCTCTATGGGCAGTCGGTGATACGAGCTGACGACARCCATG-3′. The thermal cycling conditions were 94 °C for 5 min; 35 cycles of 94 °C for 1 min, 46 °C for 20 s, 72 °C for 30 s, and a final extension at 72 °C for 7 min. Amplicons (~350 bp) were resolved on 1% agarose gels and purified using the QIAquick Gel Extraction Kit (Qiagen). Negative and positive controls were confirmed by electrophoresis but excluded from sequencing. The purified amplicons were quantified with Qubit 3.0 (Thermo Fisher Scientific), normalized to 26 pM, pooled, and sequenced on the Ion PGM™ System using the Ion PGM™ Sequencing 400 Kit and Ion 316^TM^ v2 Chip.

### Sequencing and statistical analysis

The raw sequence data were imported into *QIIME2 (v2023.2)* on the UBELIX high-performance computing cluster and processed using standardized and reproducible workflows (Supplementary Figure 2).^37^ The sequences were first inspected using the *demux* summarize plugin to evaluate per-base quality scores and read length distributions based on random base-position subsampling. Quality profile-guided truncation and filtering decisions were used to optimize denoising performance.

Adapter trimming and demultiplexing were performed using *cutadapt demux-single* (*p*-error-rate = 0) followed by primer trimming with *cutadapt trim-single* (*p*-error-rate = 0).

Denoising, quality filtering, chimera detection, and amplicon sequence variant (ASV) inference were conducted using the DADA2^38^ plugin. Specifically, dada2 denoise-single was applied with the truncation length set to 120 bp (*p*-trunc-len = 120), based on quality score decay profiles. The DADA2 workflow performs error modeling, dereplication, denoising, merging, and chimera removal using a built-in consensus approach; no additional external chimera-detection tools were applied. Reads passing DADA2 filtering thresholds were retained for downstream analyses. A feature table was generated using feature-table summarize, resulting in two core artifacts: the ASV count table (table.qza) and the representative sequence file (rep-seqs.qza).

Taxonomic assignment was performed with a pre-trained SILVA 132 classifier using *classify-sklearn*. Genus-level profiles were generated using taxa collapse. Where required, phylum names were manually harmonized to reflect the updated ICSP nomenclature to ensure consistency across taxonomic ranks.

Samples with fewer than 2,000 high-quality reads were excluded from downstream analyses to reduce sparsity bias. For multivariable modeling, ASVs were filtered by retaining features with a minimum prevalence of 10% across samples and a minimum relative abundance of 0.0001%, reducing noise from rare taxa while preserving ecological signals.

Predictive functional profiling was performed using PICRUSt2^39^ implemented via the QIIME2 plugin. ASVs were placed into a reference phylogeny, gene family abundances were inferred based on nearest sequenced taxon index (NSTI) weighting, and MetaCyc pathway predictions were generated. These functional inferences are interpreted as hypothesis-generating and not as direct measures of metabolic activity.

Statistical analyses were conducted in *R (v4.3.0)* using the packages *phyloseq,*^40^^,^^41^ *ggplot2, vegan, lda4, MaAsLin2,*^40^^,^^41^ and *SECOM.*^42^ Continuous variables were reported as the means, and group comparisons were assessed using a two-way ANOVA. Categorical variables were analyzed using the Chi-square test. A two-tailed *p*-value < 0.05 was considered statistically significant.

Correlation analysis was performed using SECOM (Sparse Estimation of Correlations for Compositional data).^42^ Because microbiome sequencing data are compositional in nature (i.e., constrained by a constant total sum per sample), naive correlation estimates can yield spurious associations driven by relative scaling rather than true biological co-variation. SECOM explicitly accounts for compositionality by incorporating sampling fraction estimation and log-ratio-based modeling, enabling more robust inference of pairwise associations in high-dimensional microbiome datasets. Correlations were estimated using the *secom_linear* function with default parameters, a Pearson correlation metric, and a threshold of |*r*| ≥ 0.3. Associations with fewer than 10 overlapping samples were excluded to minimize instability due to sparsity. Networks were constructed separately for the aspirate and biopsy datasets to avoid artificial cross-compartment correlations driven by differential total biomass or compositional structure.

Alpha diversity was calculated using Shannon and Simpson indices based on non-rarefied relative abundance data, with statistical comparisons performed using the Wilcoxon test. Sample clustering was evaluated using Principal Coordinates Analysis (PCoA). Beta diversity was calculated using BrayCurtis dissimilarities and differences in community composition were tested using PERMANOVA (*adonis2* function, vegan R package). PERMANOVA models included sample type (aspirate vs biopsy) as a fixed covariate to account for anatomical compartment effects in all secondary comparisons. In addition, PERMANOVA analyses were repeated separately for the aspirate and biopsy datasets to evaluate disease-associated compositional differences within each compartment.

Differential taxonomic abundance at the family level was assessed in relation to SIBO status and sample type using linear models implemented in MaAsLin2 (log-transformed relative abundances; significance threshold = 0.05; minimum abundance = 0.0001; minimum prevalence = 0.05). Regularized discriminant analysis (RDA) included only samples with complete metadata for SIBO status and subgroup allocation to account for the analysis variables (formula = matrix ~ SIBO + subsets). Mixed linear regression modeling of alpha-diversity trajectories following nutritional stimulation was performed using lmer (lme4 package) with the formula: Shannon ~ Time + (1|PatientID). Some analyses were additionally modeled as a continuous variable in MaAsLin2 analyses to assess linear associations between symptom burden and taxonomic abundance.

## Results

### Cohort characteristics and SIBO status

Of the 133 recruited individuals, 94 participants met the inclusion criteria and had complete duodenal culture data, forming the final study population (Figure 1 and Table 1). The cohort encompassed 9 healthy controls and 87 patients across clinical subgroups, including IBS, pre- and post-operative bariatric surgery patients, and individuals with other SIBO-associated risk factors. As expected, the preoperative bariatric group had the highest BMI values, and both bariatric subgroups contained a greater proportion of female participants than the remaining cohorts.

**Table 1.** Study population.

Categories	n	Aspirate (*n*)	Biopsy (*n*)	SIBO positive (*n*)	Gender (% male)	Age (mean +/− SD)	BMI (mean +/− SD)	IBS-SSS score (mean +/− SD)
Control	9	8	8	0 (0%)	44.4	32.2 (+/−11.6)	21.6 (+/−1.2)	35.3 (+/−57.4)
Bariatric preop	21	16	20	5 (23.8%)	23.8	44.0 (+/−13.4)	43.5 (+/−7.1)	46.0 (+/−68.3)
Bariatric postop*	16	12	15	14 (87.5%)	18.8	42.9 (+/−13.2)	27.7 (+/−6.5)	248.3 (+/−105.4)
IBS	31	23	28	16 (51.6%)	35.5	45.7 (+/−15.2)	23.3 (+/−3.8)	271.5 (+/−100.4)
Other	17	17	15	7 (41.2%)	47.1	54 (+/−17.9)	25.6 (+/−3.7)	102.5 (+/−97.2)
*p*-value (variables)					0.35	0.02	2.7E-22	2.89E-13
Missing (*n*)					0	5	8	13

*: Sleeve-Gastrectomy n = 3, Roux-Y-gastric bypass n = 13.

SIBO status was assigned based on quantitative culture of fasting duodenal aspirates. Overall, 42 participants (45%) exhibited culture-positive SIBO (>10^3^ CFU/ml), of whom 32 (34%) exceeded 10^5^ CFU/ml. None of the healthy controls demonstrated positive or intermediate culture results. The remaining 52 participants (55%) showed no evidence of SIBO (Table 2).

**Table 2.** Number of patients per aspirate culture result.

Group	SIBO culture	Negative (*n*)	Intermediary (*n*) 10^3^ –10^5^ CFU/ml	Positive (*n*) >10^5^ CFU/ml
Bariatric preop		16	1	4
Bariatric postop		2	1	13
IBS		15	6	10
Other+		10	2	5
Control		9	0	0
Total		52 (55%)	10 (11%)	32 (34%)

SIBO prevalence varied markedly across clinical subgroups. The postoperative bariatric group showed the highest burden (13/16 positive, 1 intermediate), which is consistent with known susceptibility after Roux-en-Y gastric bypass and sleeve gastrectomy. An elevated prevalence was also observed in the IBS cohort (10/31 positive, 6 intermediate) and among individuals with other predisposing conditions (5/15 positive, 3 intermediate). In contrast, the preoperative bariatric cohort had comparatively low rates (4/21 positive, 1 intermediate). These patterns highlight a strong enrichment of culture-defined SIBO in specific high-risk clinical states, particularly following bariatric surgery (Table 3).

**Table 3.** Bariatric cohort antibiotic therapy.

Group	Negative (*n*)	Intermediary (*n*)	Positive (*n*)
Bariatric postop pre-antibiotics (*n* = 26 samples of 9 patients)	2	2	22
Bariatric postop Post-antibiotics (*n* = 14 samples of 7 patients)	4	0	10
Control (*n* = 16 samples of 9 controls)	16	0	0

### Luminal and mucosa-associated communities display strong and conserved compartmentalization

From the initial 629 sequenced samples, three were excluded because of insufficient read depth. After selecting one representative sample per participant according to predefined subgroup criteria, 162 high-quality samples (76 luminal aspirates and 86 mucosal biopsies) were retained for ecological analyses.

**Figure 2.**
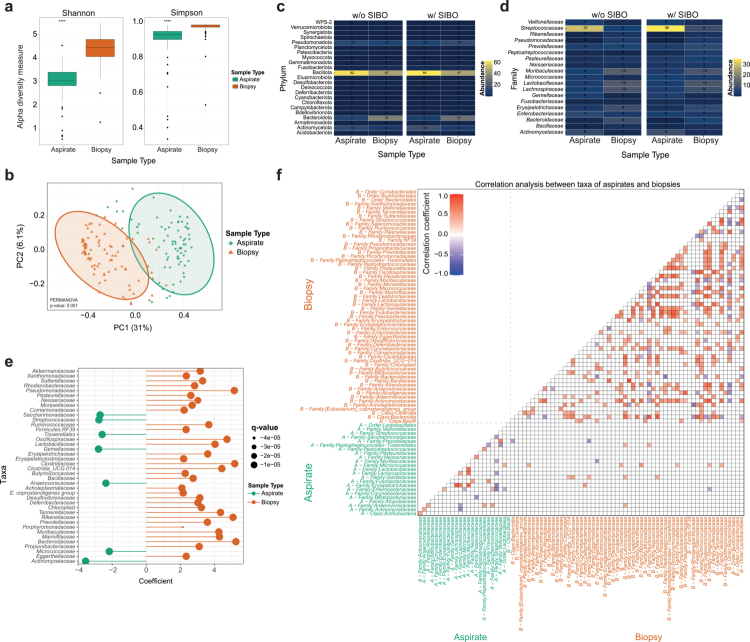
**Compartment-specific differences in duodenal microbiota composition.** (A) Alpha diversity (Shannon and Simpson indices) is consistently higher in biopsy samples compared to aspirates, indicating greater diversity within the mucosal niche. (B) Principal component analysis (PCA) based on taxonomic profiles reveals clear segregation of aspirate and biopsy samples along the primary axes of variation, demonstrating strong compartment-specific structuring. (C) Phylum-level taxonomic composition (top 20 taxa) highlights distinct community profiles between aspirates and biopsies, further stratified by SIBO status. (D) Family-level taxonomic composition (top 20 taxa) refines these differences, showing compartment-dependent shifts in dominant bacterial families across SIBO and non-SIBO conditions. (E) Differential abundance analysis identifies bacterial families significantly associated with sample type. Positive coefficients indicate enrichment in mucosal biopsies, whereas negative coefficients indicate enrichment in luminal aspirates. (F) Correlation network analysis at the family level reveals strong intra-compartmental associations within aspirate and biopsy communities, with minimal shared correlations between compartments, supporting ecological separation of luminal and mucosal microbiota.

Across all participants and clinical subgroups, we observed a pronounced and highly conserved stratification between luminal and mucosa-associated communities. Mucosal biopsies presented significantly higher alpha diversity compared with luminal aspirates (Figure 2a), which is consistent with a more structured and resource-stable ecological niche. In contrast, aspirates exhibited reduced diversity, reflecting a more selective environment shaped by intermittent substrate availability, rapid flow, and fluctuating physicochemical conditions.

Beta-diversity analyses reinforced this dichotomy: principal coordinate analysis showed near-complete separation of aspirates and biopsies along the first two components (Figure 2b). This strong clustering suggests that the duodenum hosts two fundamentally different microbial ecosystems that are conserved across individuals, clinical phenotypes, and SIBO status. PERMANOVA models, including sample type as a covariate, confirmed that the compartment significantly explained the variation in microbial composition.

At the phylum level, Bacillota dominated both compartments (Figure 2c and Supplementary Figure S3a). However, deeper taxonomic resolution revealed divergent ecological signatures. Aspirates were enriched for aerotolerant families, most prominently *Streptococcaceae*, reflecting a niche characterized by increased oxygen availability, rapid transit, and exposure to gastric acidity. These taxa are well adapted to fast-growth strategies and thrive under conditions where dispersal, rather than stable colonization, plays a major ecological role.

In contrast, mucosal biopsies harbor a broader set of obligate and facultative anaerobes, including *Lactobacillaceae, Muribaculaceae, Lachnospiraceae, Ruminococcaceae,* and *Bacteroidaceae* (Figure 2d and Supplementary Figure S3b). These communities are emblematic of biofilm-like mucosal niches where reduced oxygen tension, stable nutrient access, and continuous host-derived metabolites supporting slower, specialization-driven growth. Many of these taxa possess features such as mucin-degrading enzymes, microaerophilic metabolism, and adhesion factors that confer competitive advantages in structured environments close to the epithelial surface. Hierarchical clustering of the most relevant ASVs mirrored this strong association to sample origin (Supplementary Figure 4).

Differential abundance analysis further emphasized the ecological partitioning between compartments. Several bacterial families were significantly associated with sample type, with *Streptococcaceae, Lactobacillaceae, Gemellaceae, Bacillaceae*, and *Actinomycetaceae* enriched in aspirates, and  *Bacteroidaceae*, *Rikenellaceae, Porphyromonadaceae, Muribaculaceae, Prevotellaceae,* and *Akkermansiaceae* enriched in mucosal biopsies (Figure 2e).

The ecological network structure also differed sharply by compartment. In separate aspirates and biopsies, taxa formed tight intra-compartmental correlation modules, reflecting cohesive microbial communities shaped by the local microenvironment (Figure 2f). In contrast, cross-compartment correlations were sparse: only eight positive associations were identified between aspirate- and biopsy-derived taxa. These limited links suggest that, although the lumen and mucosa are physically adjacent, they function as largely independent microbial ecosystems with minimal synchronous fluctuations.

Notably, only two families (*Micrococcaceae* and *Anaerovoracaceae*) displayed consistent positive correlations across compartments, potentially reflecting taxa with flexible ecological strategies that enable survival in both transient and structured microhabitats. Additional selective cross-compartment links, for example, *Lachnospiraceae* in biopsies correlated with *Gemellaceae* in aspirates, may indicate niche coupling driven by metabolite exchange, host secretions, or longitudinal flow along the intestinal surface.

Taken together, these results reveal a strongly compartmentalized duodenal ecosystem characterized by distinct diversity patterns, taxonomic structures, and ecological interaction networks. The mucosa and lumen represent two sharply differentiated microbial environments within the upper small intestine: a stable, host-regulated mucosal niche, and a dynamic, flow-dominated luminal environment. This spatial structure is highly conserved across clinical phenotypes and SIBO status, underscoring its role as a fundamental organizing principle of small intestinal microbial ecology.

### Microbiota composition shows only subtle shifts with SIBO and remains stable across obesity and IBS

**Figure 3.**
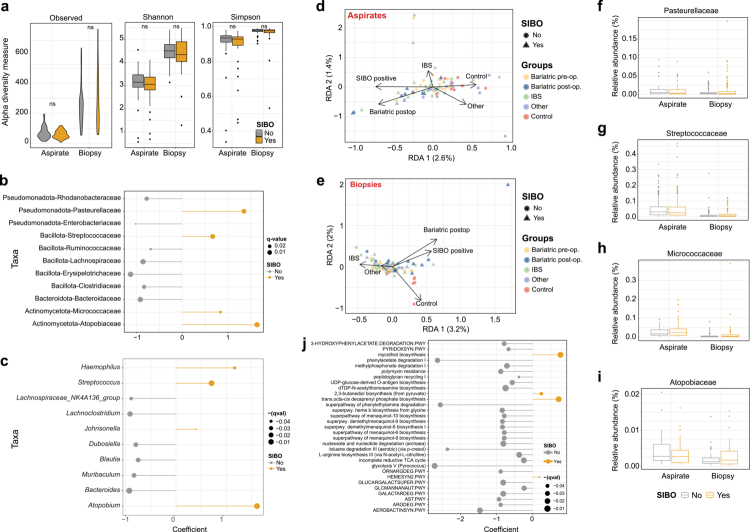
**Influence of SIBO status on duodenal microbial diversity, composition, and predicted function.** **(A)** Alpha diversity metrics, including observed richness, Shannon diversity, and Simpson diversity, are shown for aspirate and biopsy samples stratified by SIBO status. **(B)** Differential abundance analysis at the family level identifies taxa associated with SIBO status. Coefficients indicate the direction and magnitude of association, and highlighted points denote statistically significant families. **(C)** Differential abundance analysis at the genus level identifies taxa associated with SIBO status. Coefficients indicate the direction and magnitude of association, and highlighted points denote statistically significant genera. **(D)** Redundancy analysis (RDA) of aspirate samples shows that SIBO-positive samples cluster closer to the postoperative bariatric group, whereas healthy controls remain more distinct. **(E)** Redundancy analysis (RDA) of biopsy samples reveals a similar trend, with SIBO status aligning with the postoperative bariatric group and separating from healthy controls. **(F)** Abundance distribution of a representative taxon identified in the family-level differential abundance analysis, illustrating its pattern across SIBO-positive and SIBO-negative samples. **(G)** Abundance distribution of a second representative taxon from the family-level differential abundance analysis across SIBO-positive and SIBO-negative samples. **(H)** Abundance distribution of a third representative taxon from the family-level differential abundance analysis across SIBO-positive and SIBO-negative samples. **(I)** Abundance distribution of a fourth representative taxon from the family-level differential abundance analysis across SIBO-positive and SIBO-negative samples. **(J)** PICRUSt2-based pathway enrichment analysis identifies predicted functional pathways differentially associated with SIBO status.

Across both luminal and mucosal compartments, SIBO status was associated with only modest alterations in microbial community structure. Alpha diversity and species richness did not differ significantly between SIBO-positive and SIBO-negative individuals in either aspirates or biopsies (Figure 3a), indicating that bacterial overgrowth, when defined by culture thresholds, does not translate into major ecological restructuring of the duodenal microbiota.

Differential abundance analysis identified a limited set of taxa enriched in SIBO, primarily taxa from *Streptococcaceae, Micrococcaceae, Atopobiaceae,* and *Pasteurellaceae* (Figure 3b,c,f–i). These lineages are well adapted to transient, oxygen-exposed luminal conditions and likely reflect the expansion of fast-growing taxa under higher biomass states. Conversely, SIBO-negative individuals showed higher abundance of typical mucosal and anaerobe-associated families, including *Bacteroidaceae, Ruminococcaceae, Lachnospiraceae, Erysipelotrichaceae,* and *Clostridiaceae*, suggesting that classical SIBO enriches taxa suited to upper-gut aerotolerance rather than reducing overall diversity.

Redundancy analysis (RDA) supported these subtle SIBO-related shifts: SIBO-positive individuals clustered in a direction similar to that of post-bariatric surgery patients, whereas healthy controls separated in the opposite direction (Figure 3d and e). However, the variance explained by clinical factors was low (aspirates: RDA1 = 2.6%, biopsies: RDA1 = 3.2%), underscoring that SIBO and clinical status account for only a small fraction of total community variation.

**Figure 4.**
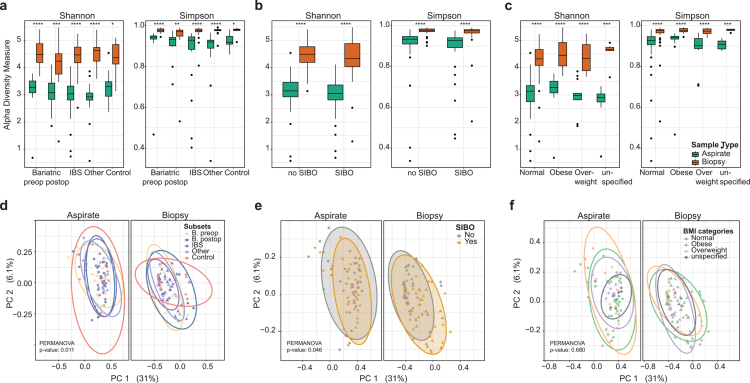
**Duodenal mucosal and luminal microbiota remain distinct across clinical subgroups.** (A) Alpha diversity, assessed by Shannon and Simpson indices, is shown for duodenal aspirates and biopsies across study cohorts, including bariatric preoperative, bariatric postoperative, IBS, other clinical groups, and healthy controls. Across cohorts, biopsy samples consistently display higher diversity than aspirates. (B) Alpha diversity, assessed by Shannon and Simpson indices, is shown for duodenal aspirates and biopsies stratified by SIBO status. In both SIBO-positive and SIBO-negative individuals, biopsy samples exhibit higher diversity than aspirates. (C) Alpha diversity, assessed by Shannon and Simpson indices, is shown for duodenal aspirates and biopsies stratified by BMI category, including normal, overweight, obese, and unspecified groups. Biopsy samples consistently display higher diversity across BMI strata. Statistical significance is indicated as ns, p > 0.05; *, p ≤ 0.05; **, p ≤ 0.01; ***, p ≤ 0.001; ****, p ≤ 0.0001. (D) Beta diversity analysis of aspirate and biopsy microbiota stratified by study cohort demonstrates clear compartment-specific clustering, with significant differences in overall community structure (PERMANOVA, p = 0.011). (E) Beta diversity analysis stratified by SIBO status identifies a modest but significant compositional effect of SIBO (PERMANOVA, p = 0.046), superimposed on the stronger separation between luminal and mucosal compartments. (F) Beta diversity analysis stratified by BMI category shows no significant effect of BMI on overall microbiota composition (PERMANOVA, p = 0.680), whereas compartment-specific separation between aspirates and biopsies remains preserved.

Beyond SIBO, obesity and IBS exerted minimal influence on the structure of the duodenal microbiota. Neither alpha nor beta diversity differed significantly across pre-bariatric, post-bariatric, obese, or IBS subgroups relative to healthy controls (Figure 4). These findings indicate that, under fasting conditions, the upper small intestinal microbiota is remarkably resilient and largely conserved across common gastrointestinal and metabolic disorders.

To determine whether these subtle compositional differences translated into functional shifts, we performed predictive metagenomic profiling using *PICRUSt2*. Overall, the functional profiles showed extensive overlap between the SIBO and non-SIBO samples, with clustering primarily driven by anatomical niche rather than the disease status (Supplementary Figure 5). Differential pathway analysis identified a limited number of pathways enriched in the SIBO samples, including mycothiol biosynthesis, 2,3-butanediol biosynthesis from pyruvate, trans-octa-cis-decaprenyl phosphate biosynthesis, and oxygen-independent heme b biosynthesis (Figure 3j). These pathways are associated with redox buffering, fermentative metabolism, and cell envelope biosynthesis, suggesting that SIBO communities may preferentially expand facultative taxa capable of maintaining redox balance and rapid growth under fluctuating oxygen conditions.

### Symptom severity reflects underlying clinical groups but shows no clear association with duodenal microbiota diversity

**Figure 5.**
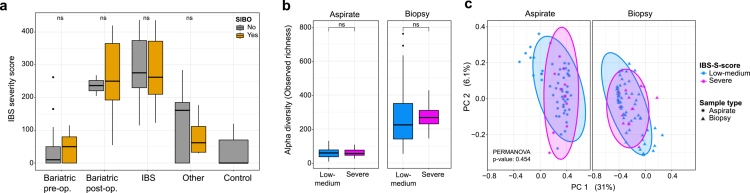
**Association between gastrointestinal symptom severity, SIBO status, and duodenal microbial diversity.** (A) IBS symptom severity scoring system (IBS-SSS) values are shown across study groups, including bariatric preoperative, bariatric postoperative, IBS, other clinical groups, and healthy controls, stratified by SIBO status. Symptom scores are highest in patients with IBS and in postoperative bariatric individuals with gastrointestinal complaints. Within each clinical group, however, SIBO status is not associated with significant differences in IBS-SSS. (B) Alpha diversity, assessed as observed richness, is compared between individuals with low-to-medium and severe IBS-SSS scores in aspirate and biopsy samples. No significant differences are detected between symptom severity groups in either compartment. (C) Beta diversity analysis of aspirate and biopsy microbiota profiles stratified by IBS-SSS category reveals no significant effect of symptom severity on overall community composition (PERMANOVA, p = 0.454).

Symptom burden, assessed by using the IBS Severity Scoring System (IBS-SSS), aligned strongly with underlying clinical subgroups. As expected, IBS patients and postoperative bariatric patients exhibited significantly higher IBS-SSS scores than preoperative bariatric patients and healthy controls (Figure 5). In the postoperative bariatric cohort, this elevation likely reflects the selection of symptomatic individuals referred for diagnostic work-up, including duodenal sampling.

Notably, SIBO status was not stratified by symptom severity. IBS-SSS scores did not differ between SIBO-positive and SIBO-negative individuals in any subgroup (Figure 5a), indicating that culture-defined bacterial overgrowth does not independently predict symptom intensity in this cohort.

We further examined whether severe symptoms (IBS-SSS > 300) were associated with measurable ecological changes in the duodenal microbiota. Neither alpha diversity nor beta diversity differed between individuals with severe versus mild-to-moderate symptoms (Figure 5b and c). These findings suggest that symptom severity is driven primarily by the underlying clinical context rather than by global shifts in duodenal microbial diversity.

In addition to threshold-based analyses, the IBS-SSS score was modeled as a continuous variable. No associations were observed with global alpha or beta diversity in either aspirates or biopsies. At the taxonomic level, a modest negative association was detected between IBS-SSS and *Ruminococcaceae* abundance (coefficient of −0.0025, *q* = 0.046), independent of SIBO status; however, the effect size was small and did not correspond to broader community restructuring.

### Antibiotic therapy shows limited short-term impact on the small intestinal microbiota

In post-bariatric patients with culture-confirmed SIBO, we compared duodenal samples obtained before and after a standardized 10-d course of selective intestinal decontamination. Across both aspirates and biopsies, antibiotic treatment did not result in significant shifts in overall microbial diversity or community composition (Supplementary Figure 6). Aspirates showed a trend toward increased alpha diversity and slightly more uniform community structure after treatment, with profiles approaching those of healthy controls, but these observations are difficult to interpret given the small sample size. Overall, these data suggest that short-term antibiotic therapy exerts limited and heterogeneous effects on the upper small intestinal microbiota. In SIBO-positive patients specifically, differential abundance analysis did not identify any taxa that were significantly different between samples obtained before and after antibiotic therapy.

### Acute luminal fat exposure rapidly increases bacterial load and transiently expands community diversity

**Figure 6.**
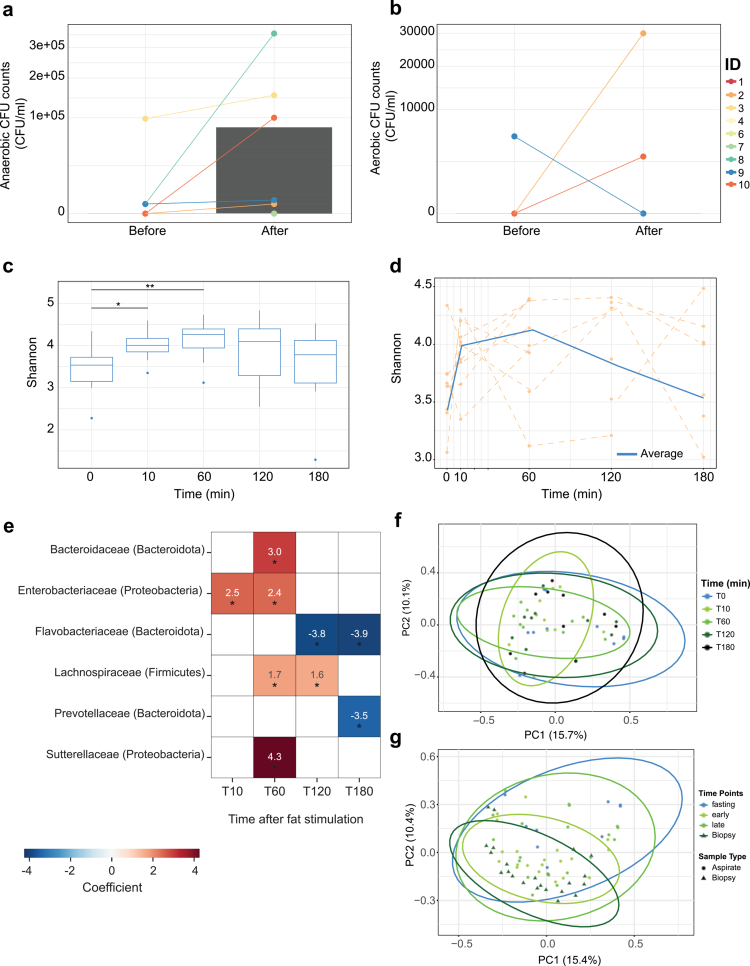
**Acute luminal fat exposure induces rapid and transient restructuring of the small intestinal luminal microbiota.** (A) Anaerobic bacterial culture counts from duodenal aspirates are shown over time in healthy lean individuals following intraluminal fat stimulation. Although baseline bacterial loads were generally low, a subset of individuals exhibited rapid bacterial blooming within 15–30 min. Individual trajectories are shown in color, and the median colony-forming unit (CFU) count is indicated in gray on a logarithmic scale. (B) Aerobic bacterial culture counts from duodenal aspirates are shown over the same time course. As observed under anaerobic conditions, fat stimulation triggered transient bacterial expansion in a subset of participants. Individual trajectories are shown in color, and the median CFU count is indicated in gray on a logarithmic scale. (C) Alpha diversity, measured by the Shannon index, is shown across serial aspirate samples collected after fat administration. Diversity increases transiently between 10 and 60 min before declining at later time points. (D) Individual Shannon index trajectories and the mean temporal profile illustrate a rapid but reversible increase in luminal diversity following nutrient exposure. (E) Heatmap of bacterial families significantly associated with time after fat administration identifies taxa contributing to the transient compositional restructuring of the luminal microbiota. (F) Principal coordinate analysis (PCoA) based on Bray–Curtis dissimilarities shows temporal shifts in aspirate community structure following fat stimulation. (G) PCoA including both aspirates and biopsies shows that post-stimulation aspirate communities transiently move toward the mucosal biopsy cluster, consistent with a temporary shift toward a more mucosa-like community configuration before partial recovery toward baseline.

In the nutrient-challenge experiment, one of ten healthy volunteers exhibited high baseline culture counts that were subsequently attributed to plate contamination and was therefore excluded. Among the remaining nine individuals, five demonstrated a clear increase in the luminal bacterial load within 30 min after intraduodenal fat administration, with median counts rising from 0 CFU/ml at baseline to approximately 10^3^ CFU/ml (Figure 6a and b). These findings indicate that nutrient availability can rapidly stimulate bacterial proliferation in the upper small intestine.

Community diversity shifted in parallel with bacterial expansion. Both Simpson and Shannon indices increased significantly in luminal aspirates over the first 60 min following fat exposure, reflecting a transient broadening of species richness (Figure 6c and d). By 180 min, diversity measures declined again, suggesting a short-lived ecological response that stabilizes as nutrients are absorbed and the local environment reverts toward fasting conditions. Together, these data show that the upper small intestinal microbiota undergoes rapid, reversible ecological restructuring in response to acute nutrient influx.

Differential abundance analysis across time points identified several bacterial families associated with the fat challenge response, including *Prevotellaceae, Enterobacteriaceae, Sutterellaceae, Bacteroidaceae, Flavobacteriaceae,* and *Lachnospiraceae*, indicating that nutrient exposure selectively expands metabolically flexible taxa in the duodenal lumen (Figure 6e).

To determine whether nutrient exposure also altered overall community structure, we analyzed beta diversity using Bray-Curtis dissimilarities. Ordination analysis revealed a transient shift in the composition of the aspirate microbiota following the intraduodenal fat challenge. The aspirated samples at intermediate time points moved closer to the mucosal biopsy cluster, then partially returned toward their fasting configuration at later time points (Figure 6f and g). PERMANOVA analysis confirmed a significant effect of time on community structure (*p*  =  0.001).

## Discussion

This study provides an integrated view of the human duodenal microbiota across anatomical niches, clinical phenotypes, and acute nutritional states. By combining paired luminal aspirates and mucosal biopsies with a controlled intraluminal fat challenge, we demonstrate that the upper small intestine harbors two sharply differentiated microbial ecosystems: a stable, host-structured mucosal community and a more selective, flow-dominated luminal assemblage. Under fasting conditions, this compartmentalization is conserved across obesity, IBS, and culture-defined SIBO, whereas acute nutrient reveals a rapid and reversible ecological response.

Consistent with colonic studies, horizontal stratification emerges as a central organizing principle of intestinal ecology.^9^^,^^43^^,^^44^ Mucosal biopsies exhibited higher alpha diversity and broader phylogenetic representation, while luminal aspirates were dominated by aerotolerant taxa, particularly *Streptococcaceae*. For the small intestine, comparable high-resolution data have been scarce,^7^^,^^13^ and our results extend prior work by demonstrating that mucosal-luminal divergence is already pronounced in the duodenum. These patterns likely reflect oxygen gradients, mucus-associated nutrient niches, and host-derived selective pressures,^10^ reinforcing the importance of anatomical sampling depth in interpreting upper small intestinal microbiome data.

Our SIBO analyses further highlight the limitations of culture-based definition. Overall species richness was conserved across the SIBO strata, and beta diversity shifts were modest, in line with earlier observations of unaltered microbial load in SIBO^30^ group and unchanged alpha diversity based on blood-agar colony counts.^28^ *Shin et al.* reported reduced diversity in mucosal biopsies but not in aspirates in SIBO-positive individuals,^7^ but we also found no FDR-significant taxonomic differences between the SIBO and non-SIBO groups.^7^ Rather than an *Enterobacteriaceae*-dominated overgrowth,^30^ we observed a predominance of *Streptococcaceae* across aspirates more broadly. Given the documented strain-level diversity and ecological flexibility of Streptococcus in the small intestine samples,^32^^,^^33^^,^^45^ this finding suggests that biomass expansion in SIBO may preferentially amplify resident aerotolerant commensals rather than induce wholesale ecological restructuring. This temporal variability contrasts with the relative stability of the colonic microbiota and aligns with the REIMAGINE data, which showed that *Streptococcaceae* were detected in virtually all duodenal luminal samples and followed an approximately log-normal distribution.^30^

Importantly, the clinical labels did not map cleanly onto the microbial structure. Although SIBO prevalence was enriched in the IBS and post-bariatric cohorts, we observed no consistent association between culture positivity and symptom severity,^6^ and only minor compositional shifts across obesity and IBS. Epidemiological and meta-analytic studies suggest increased SIBO prevalence in IBS,^25^^,^^46^^,^^47^ although much of this literature relies on heterogeneous breath testing,^47-49^ and relatively few studies have employed direct small-intestinal sampling.^23^^,^^26^^,^^50-52^ In obesity, prior studies have produced inconsistent findings;^53^ the REIMAGINE study reported increased diversity in overweight but not obese individuals,^20^ suggesting modest effect sizes requiring large cohorts or functional interrogation.^54^^,^^55^ In line with this, our fasting duodenal profiles showed no reproducible taxonomic shifts across obesity and IBS, indicating that disease-associated effects are subtle, context-dependent, or more apparent at functional or strain-level resolution. While our overall cohort is substantial for an endoscopy-based small intestinal study, stratification into individual clinical subgroups results in more modest sample sizes, and subtle phenotype-specific effects should therefore be interpreted with appropriate caution.

To place these compositional findings in a clinically relevant context, we next considered the established metabolic and host-interactive properties of the dominant taxa identified. Beyond structural organization, the taxonomic patterns observed here have potential implications for host physiology.^56^ The predominance of *Streptococcaceae* in luminal aspirates reflects enrichment of facultative anaerobes capable of rapid carbohydrate utilization and tolerance to fluctuating oxygen exposure, features that may influence local redox dynamics and short-chain organic acid production.^30^^,^^57^^,^^58^ In contrast, the mucosa-associated enrichment of *Lachnospiraceae, Ruminococcaceae*, and *Bacteroidaceae* (families linked to mucin utilization, bile acid transformation, and immunomodulatory metabolite production) suggests a functionally specialized interface with the host epithelium.^59^^,^^60^ The preservation of this mucosal architecture across disease states may therefore represent a form of ecological resilience that protects barrier function, whereas transient nutrient-driven lumenal blooms could contribute to postprandial symptoms through rapid fermentation, gas production, or altered metabolite flux.^61-64^ These observations suggest that clinically relevant perturbations in the upper small intestine may be dynamic and metabolic rather than strictly taxonomic.

Even when modeled as a continuous variable, IBS symptom severity showed no association with global diversity measures and only a minimal taxon-level signal, further showing the limited explanatory power of broad compositional metrics. These discrepancies strongly suggest that simple measures of total bacterial load or alpha diversity are unlikely to capture the mechanisms linking SIBO diagnoses and symptoms. At least three aspects are relevant. First, the taxonomic resolution of amplicon-based 16S profiling limits our ability to resolve strain-level variation that may drive clinically meaningful phenotypes. Second, the symptom burden in SIBO and IBS is likely shaped by specific pathobionts or functional capacities (e.g., pro-inflammatory, neuroactive, or metabolically disruptive outputs), rather than by global richness alone. Third, network architecture and metabolic cooperation within and across compartments may be more important than the presence of particular taxa in isolation. These considerations align with our finding that culture-positive SIBO neither predicted symptom severity nor corresponded to large-scale community restructuring. Future studies will likely require metagenomic, metatranscriptomic, or metabolomic profiling to uncover the strain-specific and functional signatures underpinning symptomatic SIBO and related syndromes.

The nutrient-challenge adds a dynamic dimension that helps reconcile relatively stable fasting profiles with the high sensitivity of the upper gut to dietary inputs. Previous work has already shown that duodenal aspirates can be sampled from healthy volunteers^65^ and that diet can rapidly reshape the fecal microbiome within days.^66^ For the small intestine, however, direct evidence has been limited. We previously demonstrated that feeding increases the small intestinal biomass within hours in ileostomy patients, providing a unique window into postprandial dynamics without repeated endoscopy.^67^ This transient convergence of luminal communities toward mucosa-associated profiles suggests that postprandial nutrient flux may temporarily reshape the physicochemical landscape of the duodenum, through changes in oxygen tension, substrate availability, and host secretions, permitting rapid expansion of facultative taxa that bridge luminal and mucosal niches. This kinetic pattern parallels the rise in ileal biomass observed 2–4 h after a standardized breakfast in the stoma model, but we additionally capture a transient diversification of the community that was not apparent in the fecal readouts. One explanation may be the use of a standardized fat-only stimulus in our protocol, which could selectively favor taxa capable of metabolizing lipid-rich substrates, thereby sharpening the temporal resolution of the response.

Methodologically, our study has several strengths: the inclusion of rigorously phenotyped healthy controls; parallel sampling of luminal aspirates and mucosal biopsies from the same duodenal site; coverage of multiple clinically relevant cohorts; and the integration of a controlled nutrient-challenge paradigm. To minimize segmental variation along the proximal small intestine, all samples were obtained from a standardized D3 location; nevertheless, subtle micro-topographical differences within the mucosal surface cannot be fully excluded.

At the same time, several limitations must be acknowledged. Clinical metadata were partly obtained through chart review, which constrained follow-up and the ability to obtain detailed treatment histories. To minimize segmental variation along the proximal small intestine, all samples were obtained from a standardized D3 location; nevertheless, subtle micro-topographical differences within the mucosal surface cannot be fully excluded. Despite using an aspiration catheter to minimize contamination, some degree of oropharyngeal carry-over cannot be fully excluded. We did not directly quantify total bacterial biomass, so relative abundance data cannot be translated into absolute taxon counts. In addition, the use of 16S rRNA gene amplicon sequencing limits taxonomic resolution and does not permit direct assessment of strain-level variation or metabolic function. To provide preliminary insight into potential functional differences, we performed predictive metagenomic profiling using *PICRUSt2*; however, these in silico pathway inferences should be interpreted cautiously and do not substitute for direct metabolomic or transcriptomic validation. Therefore, observed enrichments of specific families or genera should not be interpreted as evidence of strain-specific adaptation or functional activity. Ecological interpretations regarding nutrient utilization, mucosal adhesion, or metabolic specialization remain indirect and hypothesis-generating rather than definitive. Future studies incorporating shotgun metagenomics, metatranscriptomics, or targeted metabolomic approaches will be required to resolve strain-level heterogeneity and directly assess functional capacity within these compartmentalized communities. Finally, our culture protocol did not include MacConkey agar, which has been reported to increase sensitivity for SIBO detection and often yields higher total counts than blood agar.^28^ This is relevant because MacConkey agar primarily supports gram-negative aerobic bacilli, including Pseudomonadota (formerly Proteobacteria), whereas blood agar supports a broader anaerobic spectrum,^68^ potentially biasing culture-based SIBO definitions toward different ecological subsets.

Several questions remain. Although we identify conserved spatial architecture and nutrient-triggered ecological plasticity, future studies integrating higher-resolution techniques will be necessary to determine whether clinically meaningful functional shifts occur within taxonomically stable communities, particularly in SIBO and IBS. Disentangling compositional change from metabolic output represents a critical next step in redefining small intestinal dysbiosis beyond culture thresholds. In addition, the modest explanatory power of disease labels in our cohort highlights an unresolved question: are symptomatic states driven by total microbial biomass, specific pathobionts, network-level reorganization, or transient postprandial blooms? Addressing this issue will require longitudinal study designs incorporating absolute quantification, standardized nutrient challenges, and integration with host physiological readouts such as motility, bile acid metabolism, immune signaling, and barrier function. The pronounced divergence between mucosal and luminal communities further underscores the importance of anatomical standardization and compartment-resolved sampling in future work, as studies relying solely on luminal aspirates may overlook host-associated microbial niches that participate in immune and metabolic crosstalk. Establishing reproducible frameworks for spatially resolved small-intestinal microbiome profiling will therefore be essential for advancing both mechanistic insight and clinical translation.

Despite these limitations, our findings identify the upper small intestine as a spatially structured yet dynamically responsive microbial ecosystem. Duodenal microbiota composition remained largely stable across obesity, IBS, and culture-defined SIBO under fasting conditions, but nutrient exposure induced rapid and reproducible ecological shifts. These observations suggest that reliance on single fasting aspirates may underestimate the dynamic and spatial complexity of the duodenal microbiota and highlight the importance of spatially standardized and physiologically dynamic approaches for studying the human small intestinal microbiome.

## Supplementary Material

Supplementary Material Supplementary_Figure_3.pdf

Supplementary Material Supplementary_Figure_1.pdf

Supplementary Material Supplementary_Figure_2.pdf

Supplementary Material Supplementary_Figure_4.pdf

Supplementary Material Supplementary_Figure_5.pdf

Supplemental Material Supplementary_Figure_6.pdf

Supplementary Material Supplementary_Figures.docx

## Data Availability

The data that support the findings of this study are openly available upon publication (https://doi.org/10.6084/m9.figshare.30870725). The clinical data will be made available upon request to the corresponding authors.
