# Supplementary figures and images for: The duodenal microbiota is compartmentalized and clinically stable yet rapidly responsive to nutrient exposure
Source: Gut Microbes. 2026 Apr 18;18(1):2657053. doi: 10.1080/19490976.2026.2657053 (PMC13094253; doi:10.1080/19490976.2026.2657053)

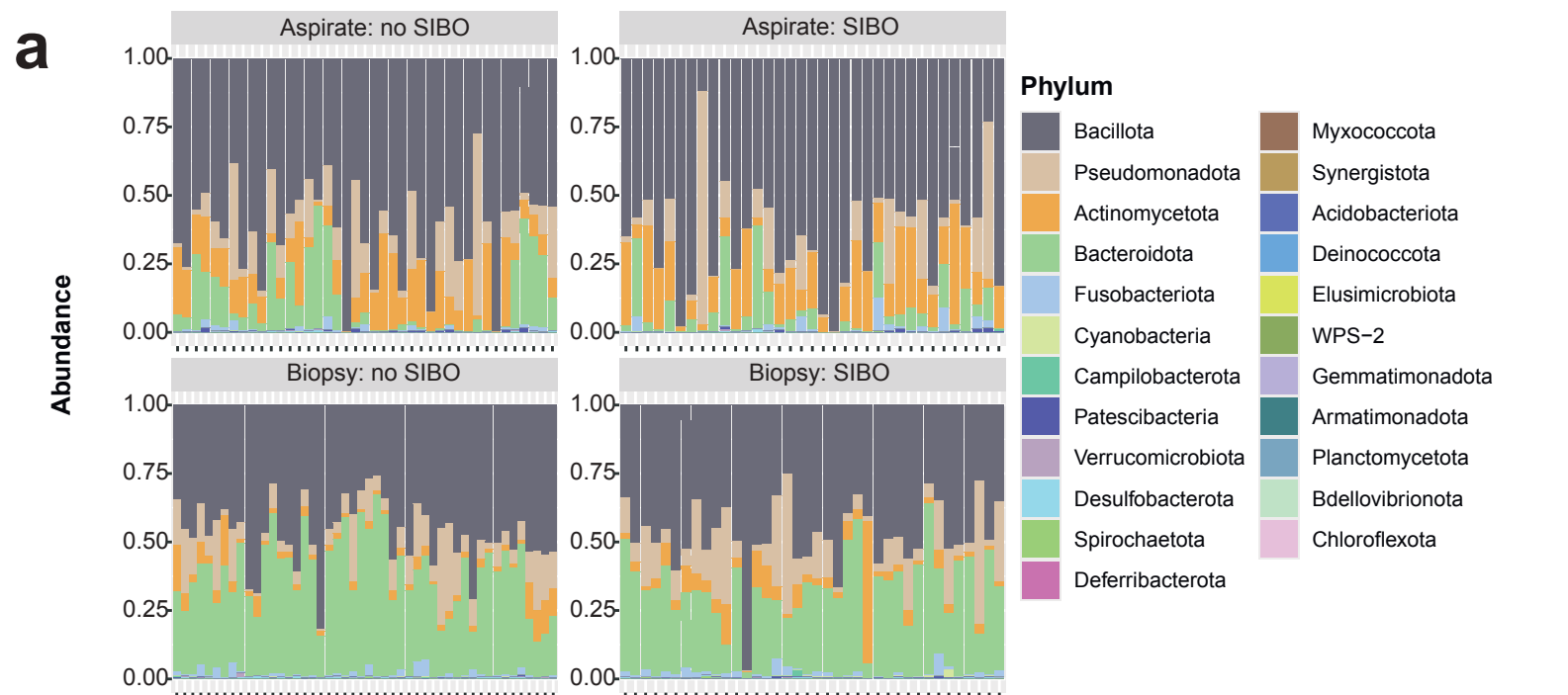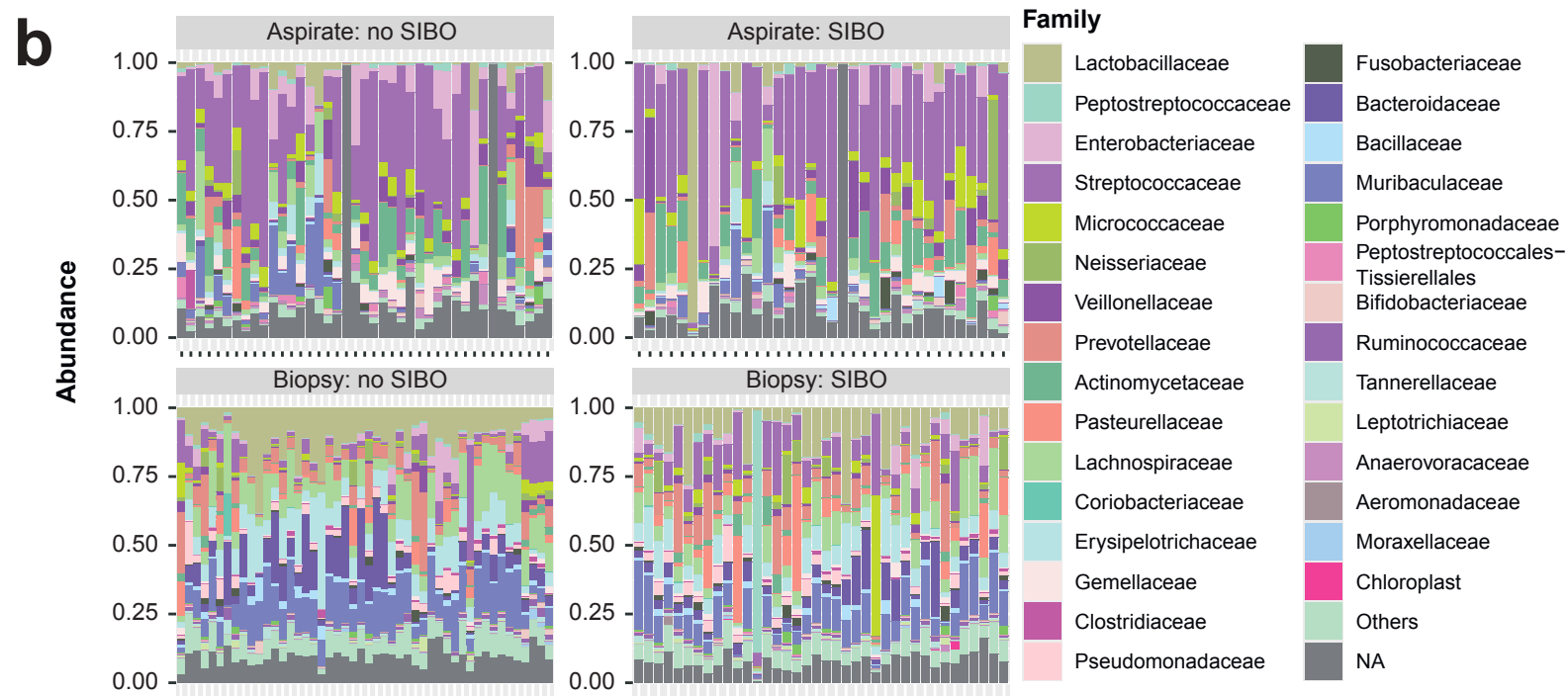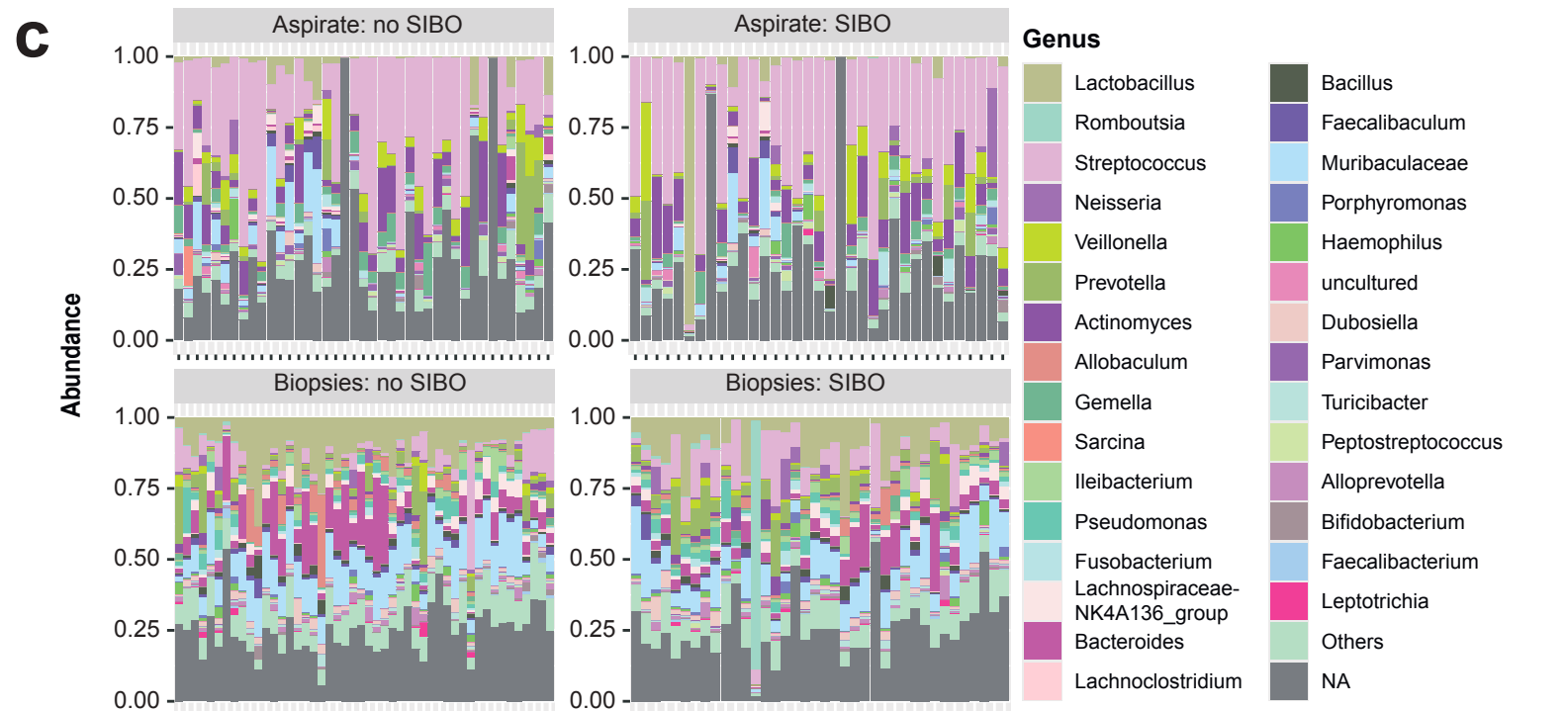

Supplement: Supplementary Material — Supplementary_Figure_3.pdf [file KGMI_A_2657053_SM6472.pdf]

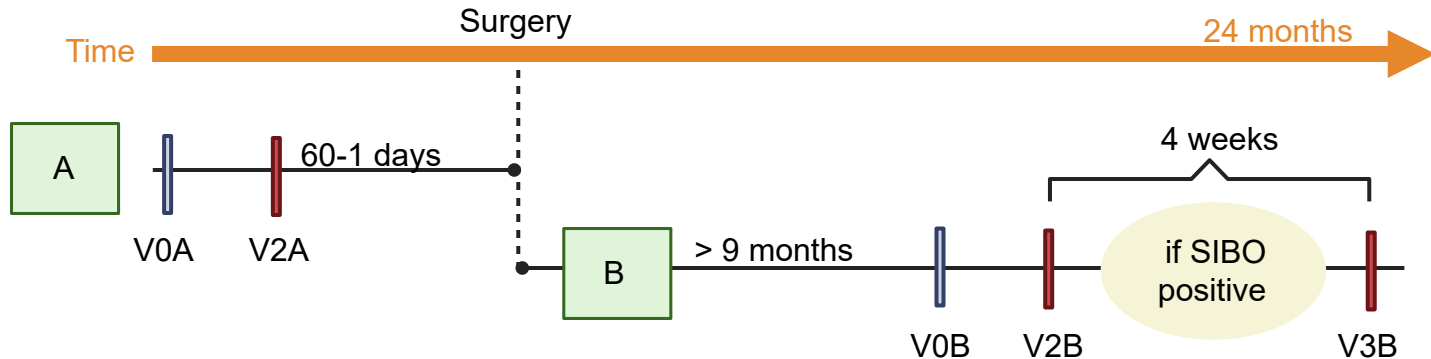

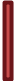 = endoscopy with sampling

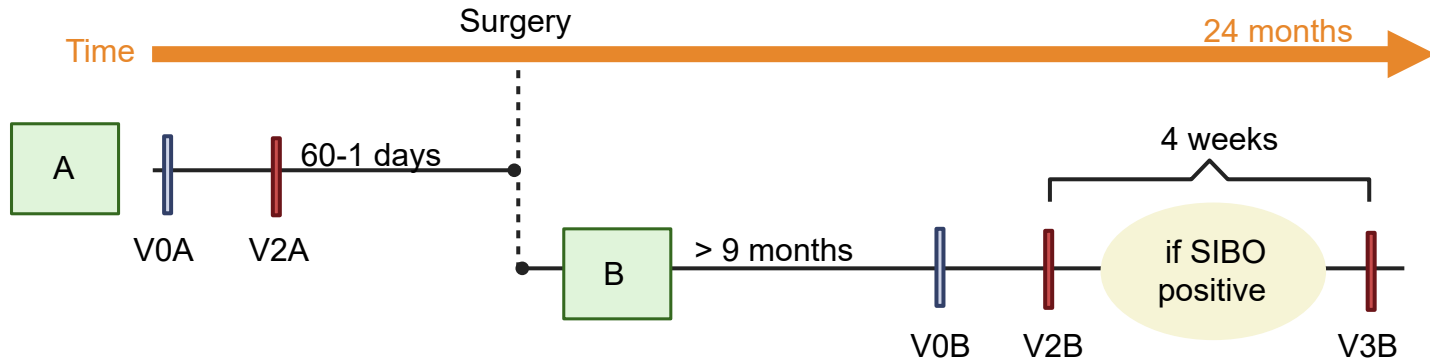

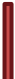 = endoscopy with sampling

Supplement: Supplementary Material — Supplementary_Figure_1.pdf [file KGMI_A_2657053_SM6451.pdf]

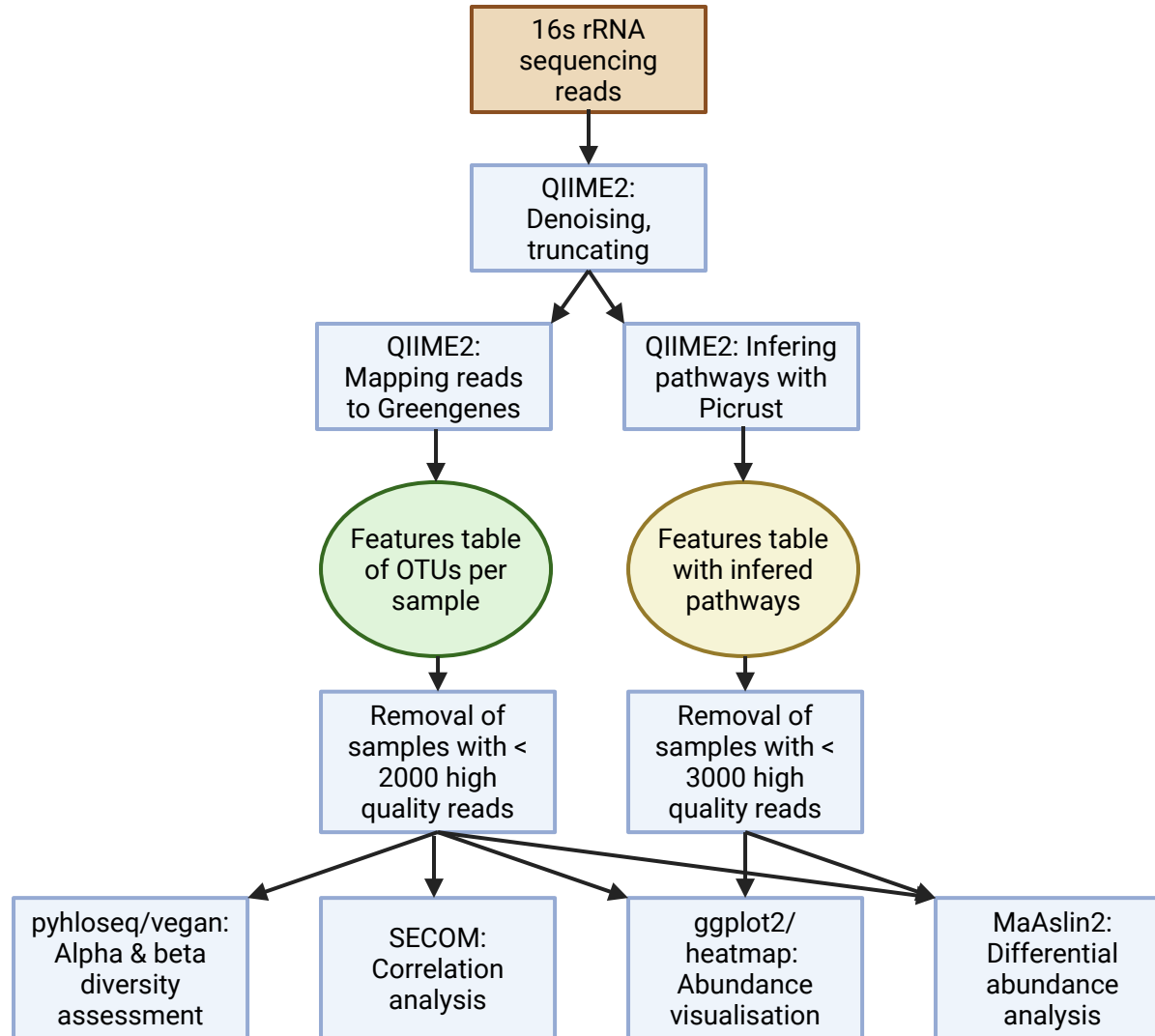

Supplement: Supplementary Material — Supplementary_Figure_2.pdf [file KGMI_A_2657053_SM6442.pdf]

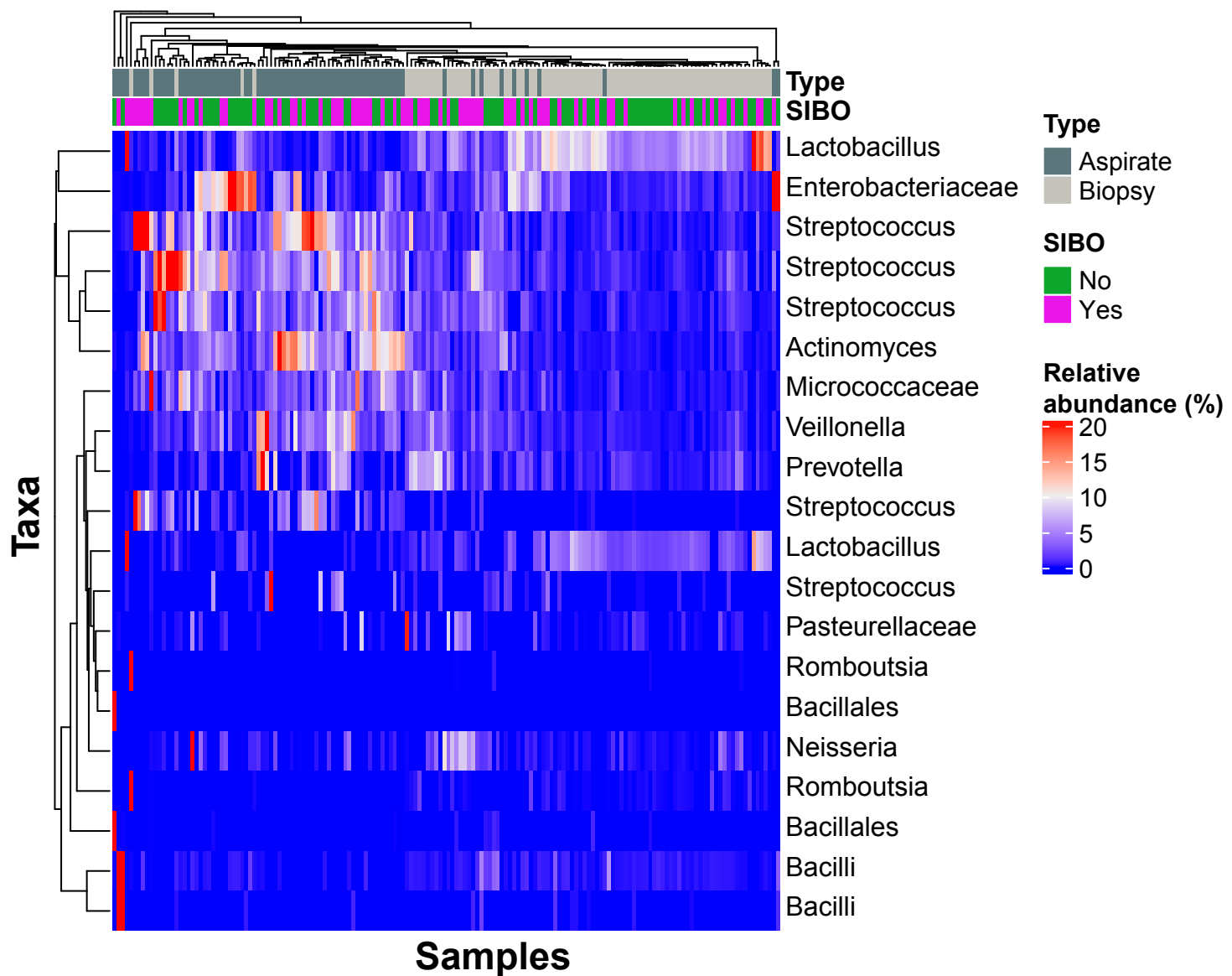

Supplement: Supplementary Material — Supplementary_Figure_4.pdf [file KGMI_A_2657053_SM6439.pdf]

**a**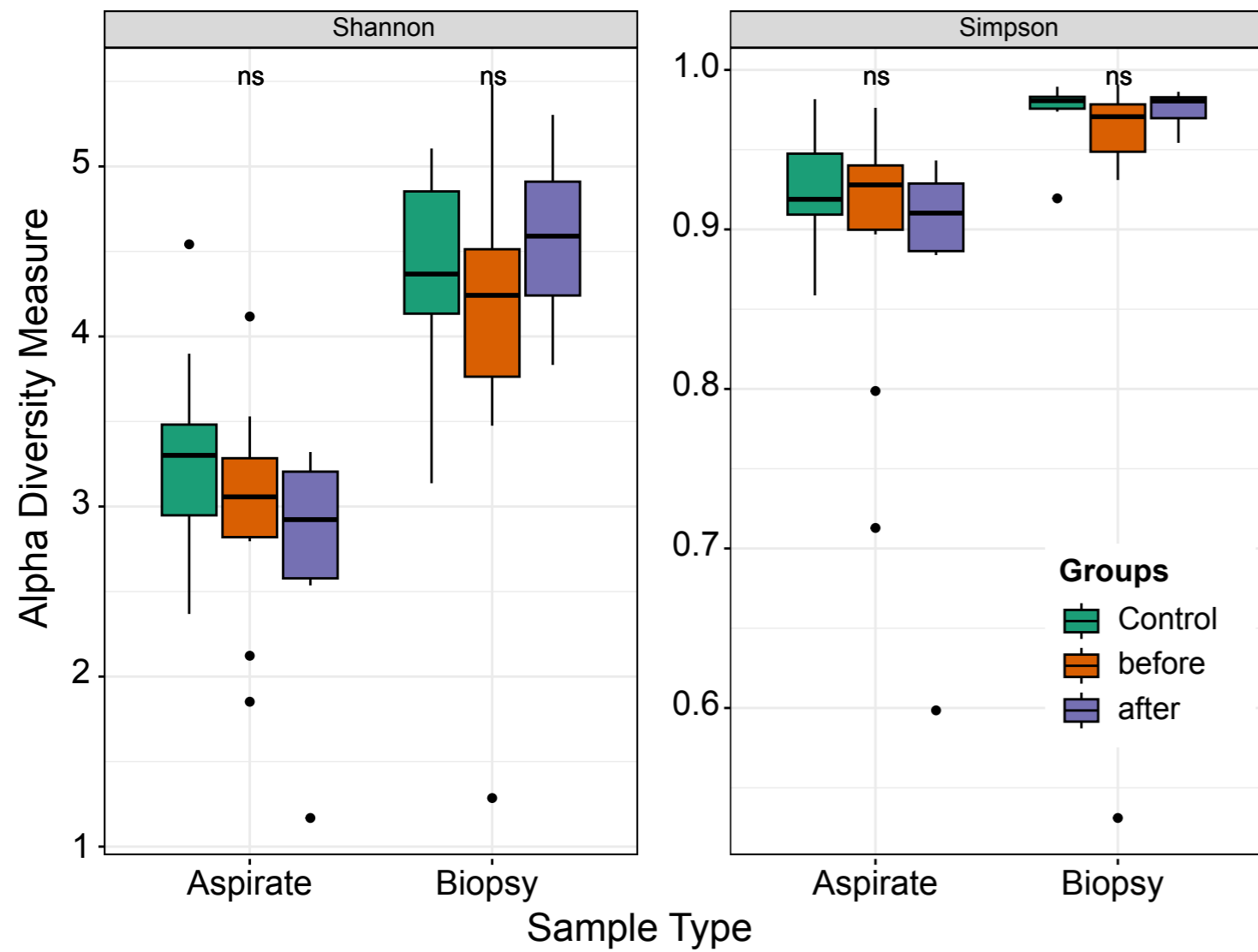**b**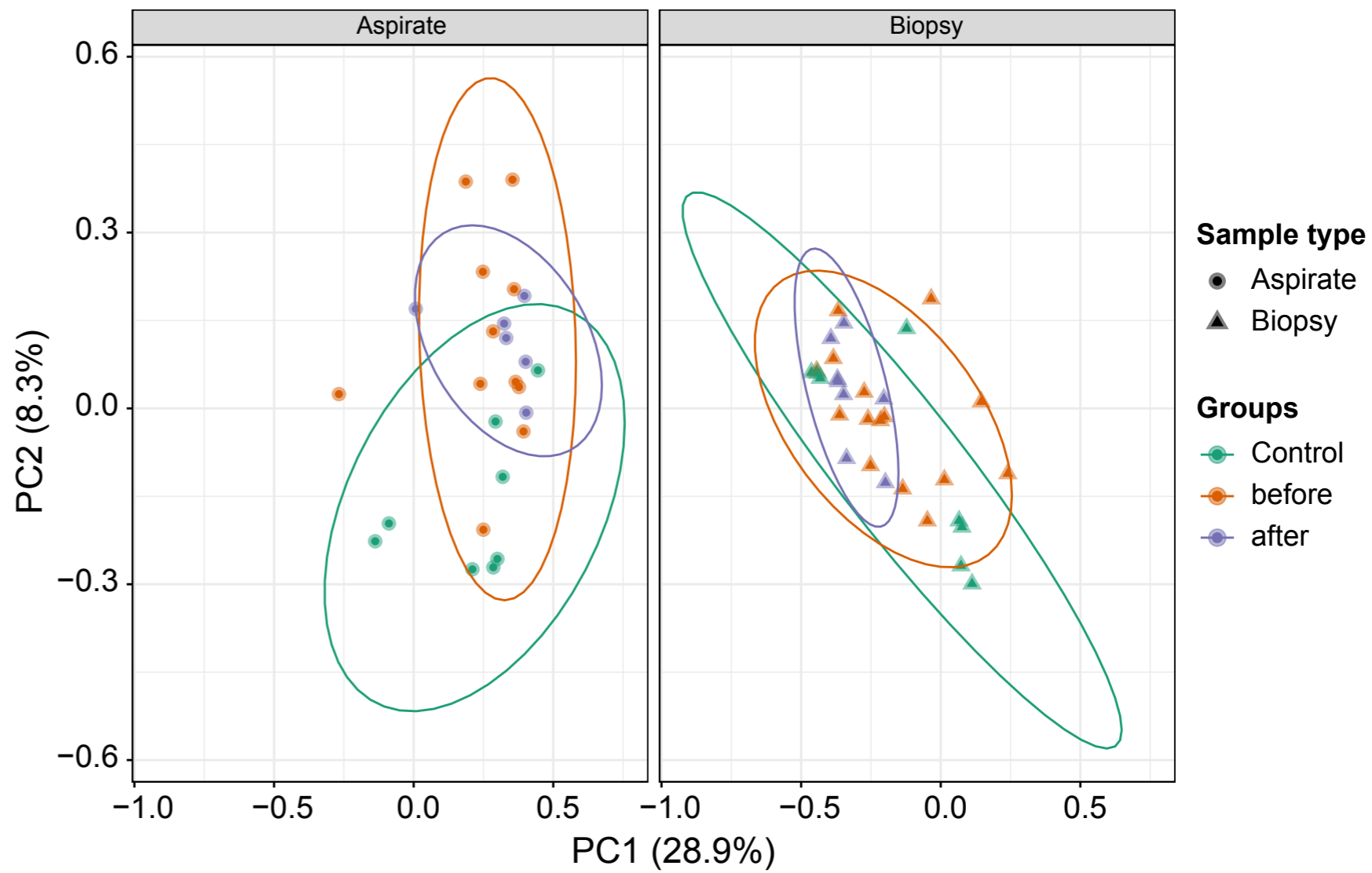

Supplement: Supplemental Material — Supplementary_Figure_6.pdf [file KGMI_A_2657053_SM6395.pdf]
