# Supplementary material for: The duodenal microbiota is compartmentalized and clinically stable yet rapidly responsive to nutrient exposure
Source: Gut Microbes. 2026 Apr 18;18(1):2657053. doi: 10.1080/19490976.2026.2657053 (PMC13094253; doi:10.1080/19490976.2026.2657053)
Supplement: Supplementary Material — Supplementary_Figure_5.pdf [file KGMI_A_2657053_SM6412.pdf]

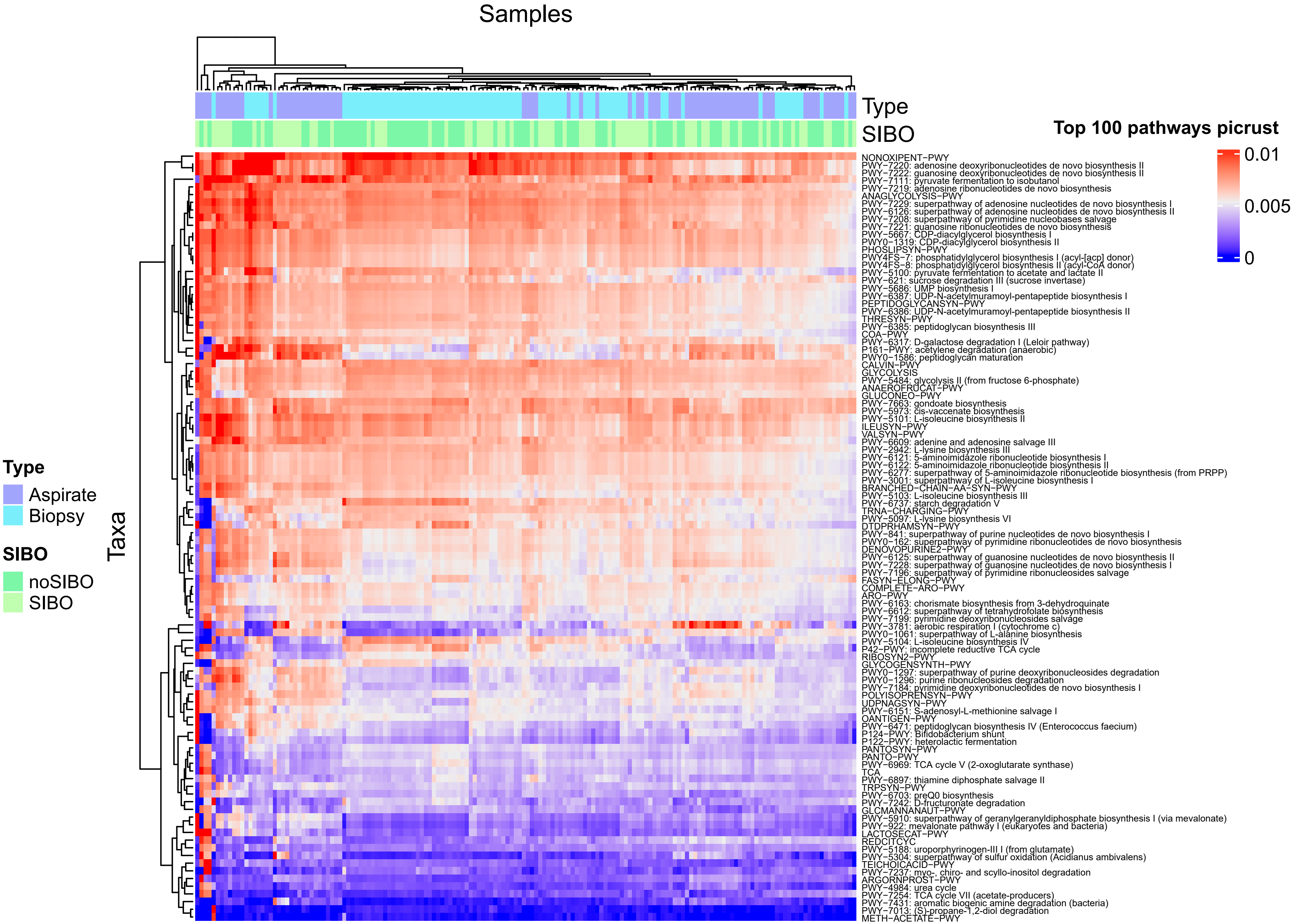
Samples
Type
SIBO
Top 100 pathways picrust
0.01
0.005
0
Type
Aspirate
Biopsy
SIBO
noSIBO
SIBO
Taxa
NONOXIPENT−PWY
PWY−7220: adenosine deoxyribonucleotides de novo biosynthesis II
PWY−7222: guanosine deoxyribonucleotides de novo biosynthesis II
PWY−7111: pyruvate fermentation to isobutanol
PWY−7219: adenosine ribonucleotides de novo biosynthesis
ANAGLYCOLYSIS−PWY
PWY−7229: superpathway of adenosine nucleotides de novo biosynthesis I
PWY−6126: superpathway of adenosine nucleotides de novo biosynthesis II
PWY−7208: superpathway of pyrimidine nucleobases salvage
PWY−7221: guanosine ribonucleotides de novo biosynthesis
PWY−5667: CDP-diacylglycerol biosynthesis I
PWY0−1319: CDP-diacylglycerol biosynthesis II
PHOSLIPSYN−PWY
PWY4FS−7: phosphatidylglycerol biosynthesis I (acyl-[acp] donor)
PWY4FS−8: phosphatidylglycerol biosynthesis II (acyl-CoA donor)
PWY−5100: pyruvate fermentation to acetate and lactate II
PWY−621: sucrose degradation III (sucrose invertase)
PWY−5686: UMP biosynthesis I
PWY−6387: UDP-N-acetylmuramoyl-pentapeptide biosynthesis I
PEPTIDOGLYCANSYN−PWY
PWY−6386: UDP-N-acetylmuramoyl-pentapeptide biosynthesis II
THRESYN−PWY
PWY−6385: peptidoglycan biosynthesis III
COA−PWY
PWY−6317: D-galactose degradation I (Leloir pathway)
P161−PWY: acetylene degradation (anaerobic)
PWY0−1586: peptidoglycan maturation
CALVIN−PWY
GLYCOLYSIS
PWY−5484: glycolysis II (from fructose 6-phosphate)
ANAEROFRUCAT−PWY
GLUCONEO−PWY
PWY−7663: gondoate biosynthesis
PWY−5973: cis-vaccenate biosynthesis
PWY−5101: L-isoleucine biosynthesis II
ILEUSYN−PWY
VALSYN−PWY
PWY−6609: adenine and adenosine salvage III
PWY−2942: L-lysine biosynthesis III
PWY−6121: 5-aminoimidazole ribonucleotide biosynthesis I
PWY−6122: 5-aminoimidazole ribonucleotide biosynthesis II
PWY−6277: superpathway of 5-aminoimidazole ribonucleotide biosynthesis (from PRPP)
PWY−3001: superpathway of L-isoleucine biosynthesis I
BRANCHED−CHAIN−AA−SYN−PWY
PWY−5103: L-isoleucine biosynthesis III
PWY−6737: starch degradation V
TRNA−CHARGING−PWY
PWY−5097: L-lysine biosynthesis VI
DTDPRHAMSYN−PWY
PWY−841: superpathway of purine nucleotides de novo biosynthesis I
PWY0−162: superpathway of pyrimidine ribonucleotides de novo biosynthesis
DENOVOPURINE2−PWY
PWY−6125: superpathway of guanosine nucleotides de novo biosynthesis II
PWY−7228: superpathway of guanosine nucleotides de novo biosynthesis I
PWY−7196: superpathway of pyrimidine ribonucleosides salvage
FASYN−ELONG−PWY
COMPLETE−ARO−PWY
ARO−PWY
PWY−6163: chorismate biosynthesis from 3-dehydroquinate
PWY−6612: superpathway of tetrahydrofolate biosynthesis
PWY−7199: pyrimidine deoxyribonucleosides salvage
PWY−3781: aerobic respiration I (cytochrome c)
PWY0−1061: superpathway of L-alanine biosynthesis
PWY−5104: L-isoleucine biosynthesis IV
P42−PWY: incomplete reductive TCA cycle
RIBOSYN2−PWY
GLYCOGENSYNTH−PWY
PWY0−1297: superpathway of purine deoxyribonucleosides degradation
PWY0−1296: purine ribonucleosides degradation
PWY−7184: pyrimidine deoxyribonucleotides de novo biosynthesis I
POLYISOPRENSYN−PWY
UDPNAGSYN−PWY
PWY−6151: S-adenosyl-L-methionine salvage I
OANTIGEN−PWY
PWY−6471: peptidoglycan biosynthesis IV (Enterococcus faecium)
P124−PWY: Bifidobacterium shunt
P122−PWY: heterolactic fermentation
PANTOSYN−PWY
PANTO−PWY
PWY−6969: TCA cycle V (2-oxoglutarate synthase)
TCA
PWY−6897: thiamine diphosphate salvage II
TRPSYN−PWY
PWY−6703: preQ0 biosynthesis
PWY−7242: D-fructuronate degradation
GLCMANNANAUT−PWY
PWY−5910: superpathway of geranylgeranyldiphosphate biosynthesis I (via mevalonate)
PWY−922: mevalonate pathway I (eukaryotes and bacteria)
LACTOSECAT−PWY
REDCITCYC
PWY−5188: uroporphyrinogen-III I (from glutamate)
PWY−5304: superpathway of sulfur oxidation (Acidianus ambivalens)
TEICHOICACID−PWY
PWY−7237: myo-, chiro- and scyllo-inositol degradation
ARGORNPROST−PWY
PWY−4984: urea cycle
PWY−7254: TCA cycle VII (acetate-producers)
PWY−7431: aromatic biogenic amine degradation (bacteria)
PWY−7013: (S)-propane-1,2-diol degradation
METH−ACETATE−PWY
