# Supplementary material for: The duodenal microbiota is compartmentalized and clinically stable yet rapidly responsive to nutrient exposure
Source: Gut Microbes. 2026 Apr 18;18(1):2657053. doi: 10.1080/19490976.2026.2657053 (PMC13094253; doi:10.1080/19490976.2026.2657053)
Supplement: Supplementary Material — Supplementary_Figures.docx [file KGMI_A_2657053_SM6383.docx]

**SUPPLEMENTARY FIGURES**

**Supplementary Figure 1. Study procedures in bariatric cohorts.** V: visit. Morbid obese patients (BMI > 35 kg/m^2^) were recruited either prior to (A) or after bariatric surgery (B). Pre-operative bariatric patients underwent routine upper endoscopy at study visit V2A within two months prior to their scheduled bariatric surgery. Post-operative patients underwent upper endoscopy at visit V2B as part of their clinical evaluation for suspected SIBO. All endoscopic procedures were conducted within 14 days of study enrollment (V0A/B). If SIBO was confirmed in Group B by endoscopy-derived luminal aspirate culture, patients received a 10-day course of selective intestinal decontamination with gentamicin/polymyxin capsules. A follow-up endoscopy was performed within two weeks after completing the antibiotic regimen at study visit V3B.

**Supplementary Figure 2. Bioinformatic workflow for 16S rRNA gene analysis.** Overview of the analytical pipeline from raw sequencing reads to downstream statistical analyses. Reads were processed in QIIME2 (v2023.2) for quality filtering, denoising (DADA2), and taxonomic assignment using a pre-trained SILVA classifier. Feature tables were generated for taxonomic profiling and predictive functional inference using PICRUSt2. Samples below predefined read-depth thresholds were excluded prior to analysis. Downstream analyses included alpha and beta diversity assessment (phyloseq, vegan), differential abundance testing (MaAsLin2), compositionality-aware correlation analysis (SECOM), and data visualization (ggplot2).

H

**Supplementary Figure 3**. **Taxonomy of profile per individual sample.** Bar plot shows microbiota profile for aspirates and biopsies with and without SIBO at (a) phylum, (b) family and (c) genus level.

**Supplementary Figure 4**. **The top 20 most abundant ASVs are depicted by their best available taxonomic annotation.** Unsupervised hierarchical clustering of the top 20 ASVs shows an almost perfect clustering according to sample type (grey bars on top), whereas SIBO status does not exhibit a clear clustering (pink bar: SIBO positive, green bar: SIBO negative). Included in the top 20 ASV are several separately annotated ASVs assigned to the *Streptococcus* genus, indicating additional diversity on the species level not resolved by the current analysis method.

**Supplementary Figure 5. Predicted functional pathway profiles of the duodenal microbiota inferred by PICRUSt2.** Heatmap showing the relative abundance of the top 100 predicted metabolic pathways across duodenal samples.

**Supplementary Figure 6. Impact of antibiotic treatment on small intestinal microbiome.** Effect of selective intestinal decontamination on small intestinal microbial diversity in post-bariatric patients with culture-confirmed SIBO. (A) Alpha diversity (Shannon index) comparing pre- and post-antibiotic samples in both luminal aspirates and mucosal biopsies, with healthy controls shown for reference. (B) Beta diversity analysis based on Bray-Curtis dissimilarity visualized by principal coordinates analysis (PCoA).
